# The Role of Benson's and Mitchell's Relaxation Techniques in the Management of Premenstrual Syndrome: An In-Depth Review

**DOI:** 10.7759/cureus.43822

**Published:** 2023-08-20

**Authors:** Anushka Raipure, Shubhangi Patil

**Affiliations:** 1 Department of Community Health Physiotherapy, Ravi Nair Physiotherapy College, Datta Meghe Institute of Higher Education and Research (DU), Wardha, IND

**Keywords:** quality of life, premenstrual syndrome, pain, mitchell’s relaxation technique, benson relaxation technique

## Abstract

Premenstrual syndrome (PMS) is a menstrual disorder characterized by physical, behavioral, and emotional symptoms typically occurring during the luteal phase. These symptoms are usually mild and somatic and often subside during or just before the start of menstruation. Medical professionals employ pharmacological and non-pharmacological treatments as the primary approach to managing PMS. One effective non-pharmacological method is Laura Mitchell's physiological relaxation treatment, which has been shown to enhance immunological function, reduce depression, and improve the overall quality of life. Another beneficial technique is Benson's relaxation technique, established by Herbert Benson in 1970, providing a simple yet powerful stress-release strategy. For this study, research papers were searched using various reputable databases such as PubMed, Scopus, Web of Science, and Google Scholar websites, with data collected up to the end of 2021. The publication dates of the review articles were not restricted, allowing for a comprehensive overview. However, it is important to note that only a limited number of accessible studies have been found, underscoring the need for further research. Future investigations should focus on high-quality evidence with reliable outcome measures to draw more conclusive results about which relaxation technique offers better relief for PMS.

## Introduction and background

Premenstrual syndrome (PMS) is a group of symptoms that occur during the luteal phase of the menstrual cycle and causes severe distress and impairment in functional capacity [1]. In 20% to 32% of cases, premenopausal women are affected by PMS, which refers to various physical and emotional symptoms that occur in the days leading up to menstruation [2]. The luteal period of the menstrual cycle primarily affects young and middle-aged women, causing a recurring condition with pronounced physical and mental manifestations [3]. Although the cause of many illnesses is unknown, research shows that abnormal neurohormone and neurotransmitter control is involved [4]. Depression, mood swings, stomach pains, breast soreness, headaches, and weariness seem to be just a several of the complaints that may manifest [5].

Relaxation methods are therapeutic activities that help people reduce stress and anxiety physically and psychologically [6]. Relaxation techniques have long been a staple of psychotherapy; nevertheless, they may be used as supplementary treatments in various healthcare settings to treat patients suffering from multiple ailments, mainly but not confined to anxiety, depression, pain, and stress [7]. Relaxation methods are psychophysiological treatments that relieve stress by promoting physical and cognitive distension [8]. These approaches are commonly utilized in physiotherapy and efficiently reduce anxiety and suffering [9]. Although relaxation is a well-known and often-used concept, a precise definition of clinical relaxation seems elusive [10].

Laura Mitchell's physiological relaxation treatment improves immunological function, reduces depression, and improves the overall quality of life [11]. Mitchell's muscular relaxation technique uses diaphragmatic breathing and is premised mainly on the reciprocal inhibitory principle of physiology [12]. The opposite set of muscles relaxes when one group of forces operating on the joint works. Laura Mitchell's relaxation method utilizes reciprocal relaxation, wherein she moves one portion of the body in the opposite direction of a tense area and then releases it [13].

Herbert Benson (1970) established Benson's relaxation technique as a simple strategy for releasing stress [14]. This strategy resulted in improvements in sleep quality, overall quality of life, and a reduction in pain intensity. Concurrently, it diminished feelings of anxiousness and mood disturbances and fostered physical activity [15]. Benson's technique predominantly focuses on inducing physical relaxation, effectively mitigating various physiological stressors. Importantly, Benson's relaxation technique confers several benefits, possesses user-friendly attributes, and has no adverse effects on individuals [16]. This practice is said to give a reprieve from the physical and psychological impacts of worry and stress [17].

Mitchell's relaxation technique

Mitchell's relaxation method is a standardized stress management technique widely used, especially in obstetrics and gynecology [18]. Mitchell's physiological relaxation method incorporates diaphragmatic breathing exercises and a sequence of serial isotonic contractions focused on reciprocal inhibition [19]. According to Laura Mitchell (1977), the method produces postural realignment by reversing the "punching stance," a stress-related posture [20]. If left untreated, epinephrine and norepinephrine are released, causing the adrenal and lymphatic glands to grow, resulting in physical sickness and mortality [21]. This relaxation approach works by triggering the relaxation response, which helps to restore nervous system imbalances. Hormones are released, which have a wide-ranging influence on the cardiorespiratory system, causing this reaction [22].

Ferreira and Kulkarni's investigation showed that meditation with visualization and Mitchell's relaxation method was beneficial in lowering the degree of exhaustion and headaches in PMS. The investigation established that Mitchell's method of relaxation technique substantially reduced the intensity of fatigue and headaches in individuals with PMS compared to meditation with visualization [23]. Mitchell's basic physiological relaxation approach revealed extremely substantial decreases in pain intensity, pulse rate, respiration rate, and TG MYO feedback 420v, according to research by Kosery et al. As a result, researchers discovered it to be an effective, non-invasive, safe, inexpensive, and simple therapeutic strategy for relieving discomfort and stress associated with primary dysmenorrhea [24].

Laura Mitchell's physiological relaxation approach, according to Shareinia et al., is more helpful in reducing pain severity. Still, Jacobson's progressive relaxation strategies enhance the quality of life in menstrual pain. Both approaches can be used in healthcare settings to reduce symptom severity, promote quality of life, and reduce absenteeism and stress. College students can greatly benefit from incorporating these practices into their lives to enhance their reproductive well-being and overall quality of life. These methods are easily accessible, affordable, and free from harmful effects, making them an ideal choice for students seeking to improve their well-being. By embracing these techniques as regular practices, students can experience notable physical and emotional health improvements, contributing to a more balanced and fulfilling college experience [25].

Benson's relaxation technique

Benson's meditation is a primary relaxation method that can help block sympathetic nervous system hormones. This blocking can help break the cycle of anxiety and alleviate the symptoms that come with it [26]. It comprises five parts: 1) a quiet atmosphere: selecting a location that is free of distractions; 2) adopting a relaxing posture, such as seated, standing, sleeping, or walking, is essential; 3) suitable acceptance: relaxing all the muscles commencing from the soles of the feet up to the facial muscles, ensuring that everything is relaxed; 4) concentration: concentration and staying oriented to the breathing patterns and inspiring through the nose, with expiration through the mouth; 5) suitable acceptance: maintaining relaxed attitude [27]. The methods act by lowering the sympathetic nervous system's activity and reducing the levels of endogenous catecholamine [28]. It causes muscular relaxation and a decrease in various emotions like tension, anxiety, and sadness. The relaxation approach of Benson is also linked to an increase in the patient's self-esteem [25]. Individuals may control their breathing, lower their heart rate and blood pressure, and avoid many harmful physiologic responses to stress by concentrating [29]. Individuals situate themselves properly with closed eyes to execute Benson's relaxing method. They focus on gradually relaxing their muscles, beginning at the soles of their feet and making their way upward to the tops of their heads. They inhale with their nose and exhale gently through their mouth, keeping their muscles calm and conscious of their breathing. They repeat "one" to themselves as they exhale and proceed to breathe freely and smoothly [30]. After 20 minutes of these activities, they sit quietly for several moments with their eyes closed (and then opened) [31].

A quasi-experimental study by Olia et al. concluded that relaxation responses help treat symptoms of a range of stress-related diseases, including PMS. A decrease in oxidative mediators and reactive species, and an increase in antioxidant capacity, accompanied Benson's relaxation. These changes reduced the adverse effects of stress and alleviated psychosomatic symptoms in people with PMS. The study's findings highlighted the usefulness of Benson's relaxation as a supplemental therapy program for reducing oxidative marker levels and physical and cognitive symptoms in people with PMS [32].

Over five months, Goodale et al. evaluated the impact of the muscle relaxation response on PMS in 46 women. The researchers assigned the participants to one of three groups based on their responses: documenting, studying, or relaxing. The relaxation response group benefited significantly more than the charting and reading groups regarding physical complaints [33]. According to Hassan Ahmed et al. quasi-experimental study, the intensity of PMS within Benson's relaxation therapy group decreased significantly over time (Fr test=34.696; p=0.001). Benson's relaxation treatment regularly is an excellent technique for achieving sympathetic and parasympathetic system equilibrium. Relaxing the body reduces the physical consequences of stress, calming the mind. This process can help break the anxiety cycle and alleviate the accompanying symptoms. This blocking can help break the cycle of fear and alleviate the symptoms that come with it [34].

## Review

Objective

Our primary objective was to provide a comprehensive summary of the relevant literature concerning the effects of relaxation techniques on pain and quality of life in patients with PMS. By diligently examining a wide array of studies and incorporating a diverse range of research methodologies, we aimed to shed light on these techniques' potential benefits and impact on the well-being of individuals dealing with PMS. Through this meticulous review, we sought to offer valuable insights and evidence to advance the understanding and potential therapeutic applications of relaxation techniques in managing premenstrual symptoms and enhancing the overall quality of life for affected individuals.

Study design and setting

This review study comprises extensive quantitative research, examining the effects of relaxation techniques in alleviating PMS. By critically analyzing a substantial body of quantitative data, we aimed to provide a comprehensive understanding of the potential benefits and efficacy of relaxation techniques as therapeutic interventions for managing premenstrual symptoms. Through this rigorous investigation, we endeavor to contribute significantly to the existing body of knowledge, offering valuable insights into the role of relaxation techniques in enhancing the well-being and quality of life of individuals affected by PMS.

Data sources and search engines

This comprehensive review incorporated diverse studies, including original articles, systematic reviews, meta-analyses, and randomized control trials. A combination of carefully chosen keywords and MeSH terms was employed to ensure comprehensiveness in identifying relevant articles. The screening process involved strategically using keywords to refine the article selection. The chosen keywords encompassed crucial aspects such as pain, PMS, relaxation techniques, Mitchell's relaxation technique, Benson's relaxation technique, and quality of life. Employing reputable online search engines such as PubMed, Scopus, Web of Science, and Google Scholar, we curated a comprehensive collection of articles to accumulate valuable information. This meticulous approach sought to establish a robust foundation for the review, facilitating a profound understanding and exploration of the subject matter.

Inclusion criteria

The review encompassed studies that met specific inclusion criteria aligned with the focus of the research. These criteria dictated that the studies under consideration pertained to individuals afflicted by PMS and were conducted within a spectrum of clinical environments, including hospitals, laboratories, and relevant rehabilitation centers. The review comprehensively integrated diverse research methodologies, encompassing randomized controlled trials (RCTs)and observational studies, all interrogating various facets of the intricate PMS landscape. We maintained stringent adherence to studies involving human subjects, published comprehensive full-text articles within esteemed peer-reviewed medical journals, and articulated proficiently in English. This meticulous and judicious selection process was systematically orchestrated to ensure the assimilation of pertinent and methodologically robust research articles, thereby augmenting the scholarly exploration of PMS.

**Figure 1:**
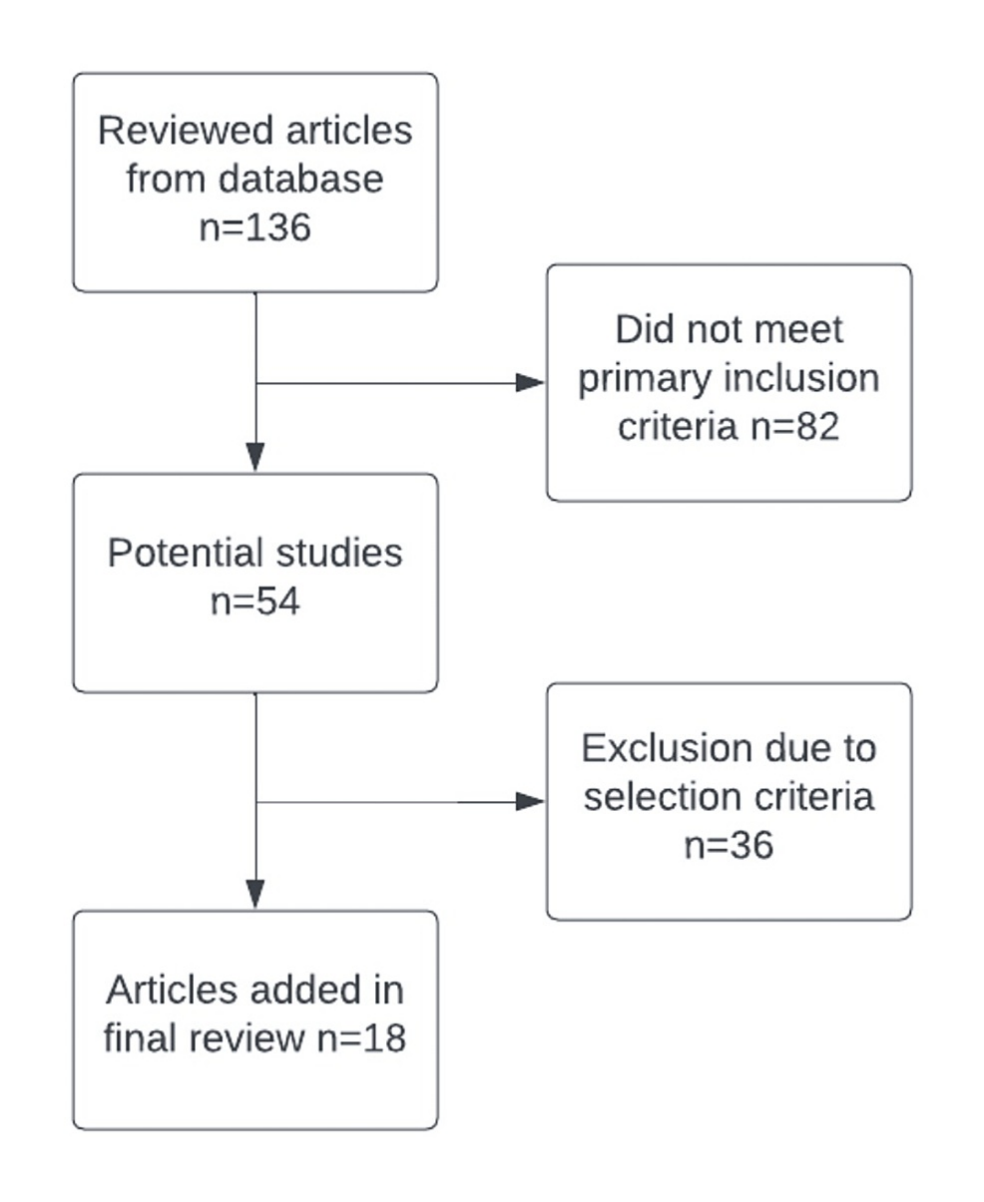
Flowchart of the article selection process.

The initial search yielded 136 relevant articles. After meticulously examining the references, we successfully identified 54 additional articles. Subsequently, we rigorously applied stringent inclusion criteria, carefully accounting for factors like the unavailability of full text or language barriers. The process ultimately led to the inclusion of 18 highly relevant articles in the study. This meticulous selection ensured that only the most pertinent and high-quality studies were incorporated, contributing to the study's robustness and reliability (Figure 1).

Exclusion criteria

The inability to access the full version of articles emerged as one of the primary reasons for their exclusion. This obstacle significantly impacted the study's selection process, necessitating careful consideration.

Table 1 shows the characteristic information of the articles included in the review.

**Table 1:** Characteristics of key references. CABG, coronary artery bypass graft; CAG, coronary angiography; PCI, percutaneous intervention; PMT, premenstrual tension; DASS, Depression Anxiety Stress Scales

Sr. no	First author	Year published	Year conducted	Study location	Subject/sample	Type of study	Results
1	Ganesh et al. [13]	2017	2017	Belgaum	34 individuals with dysmenorrhea	Comparative interventional study	In patients with primary dysmenorrhea, Laura Mitchell's relaxation techniques proved beneficial in reducing the severity of pain.
2	Molazem et al. [15]	2021	2018	Shiraz, Iran	80 hemophilia patients	Clinical trial study	The study concluded that Benson's relaxation helped reduce pain intensity in the patients.
3	Mohamed et al. [19]	2015	Unclear	Cairo	30 females	Comparative interventional study	Mitchel's relaxation was effective in treating PMT.
4	Ferreira et al. [23]	2018	Unclear	Ahmednagar	30 normal healthy female individuals	Comparative interventional study	Their study concluded that Mitchell's relaxation technique was very effective in individuals with premenstrual syndrome, especially with fatigue and headache
5	Kosery et al. [24]	2006	Unclear	Cairo	25 females	Interventional study	The study concluded that Mitchell's method was beneficial in lowering pain intensity and tension from primary dysmenorrhea.
6	Zenouzi et al. [28]	2021	2015-2016	Iran	70 pregnant women	Randomized clinical trial	The study proved Benson's technique efficiently reduced DASS-21 scores in the individuals.
7	Mirhosseini et al. [29]	2021	2017-2018	Kashan, Iran	60 multiple sclerosis patients	Randomized controlled trial	According to the study's findings, Benson's technique was affordable and was conspicuous in improving social, physical, mental, and general health status in multiple sclerosis patients.
8	Daneshpajooh et al. [30]	2019	2017-2018	Iran	132 burn patients	Randomized controlled trial	The study concluded that Benson's technique and aromatherapy effectively tapered pain anxiety in patients suffering from a burn.
9	Habibollahpour et al. [31]	2019	2016-2017	Qazvin, Iran	80 elderly patients	Randomized clinical trial	The study emphasized that Benson's relaxation technique provides an efficient and affordable method of evoking sleep and improving sleep quality in community-dwelling geriatric individuals.
10	Olia et al. [32]	2019	Unclear	Khoy, Iran	30 female nursing students	Quasi-experimental study	The study significantly proved the utilization of Benson's relaxation for lowering mental and physical symptoms and varying levels of oxidative stress markers.
11	Ahmed et al. [34]	2022	Unclear	Egypt	100 female students	Quasi-experimental study	The study concluded that pilates was more effective in reducing premenstrual syndrome symptoms than Benson's relaxation technique.
12	Kamalifard et al. [35]	2015	Unclear	Indonesia	60 post-cesarean section females	Quasi-experimental study	The study found that the Benson technique effectively lowered pain intensity in women after cesarean section.
13	Jose et al. [36]	2016	Unclear	England	201 patients with fibromyalgia	Randomized control trial	Mitchell's relaxation technique proved valuable in lowering the pain problems in sleep and fatigue.
14	Mowla et al. [37]	2017	Unclear	Tehran, Iran	100 parents having at least one child suffering from chronic disease	Quasi-experimental study	Benson's relaxation method improved the parents' quality of life.
15	Poorolajal et al. [38]	2017	2014	West Iran	144 Patients undergoing CABG, CAG, PCI, or general surgery	Single-masked, randomized control trial	The therapy effectively reduced preoperative anxiety and hemodynamic status, namely blood and pulse pressure.
16	Heshmati et al. [39]	2020	Unclear	Sabzevar, Iran	65 hemodialysis patients	Single-masked, randomized, parallel-group, control trial	Benson's relaxation technique significantly enhanced the daily living activities of the patients on hemodialysis.

Discussion

PMS is a significantly prevalent condition, with its symptoms affecting more than 90% of menstruating women. It manifests as a collection of physiological, intellectual, psychological, and behavioral symptoms that cyclically arise and fade throughout the luteal phase of the normal reproductive cycle [35]. Relaxation techniques serve as a valuable means to alleviate the adverse effects of stress and aid in managing stress-related health issues. Individuals can easily acquire these fundamental relaxation practices and access them at no cost or a nominal expense. Despite the widespread utilization, a substantial body of evidence to definitively validate the efficacy of additional treatments that are non-drug based in managing PMS remains conspicuously absent [37].

Mitchell's method is an aural relaxation technique done alone and quietly. It emphasizes the mind-body psychoneuroimmunological connection, includes guided imagery, relaxation techniques, and deep breathing, and implies complete involvement and autonomy. The stress-related posture is hypothesized to promote muscular stiffness and dystonic patterns, increase muscle tension, and alter the neurological and endocrine systems [40].

Benson's technique is straightforward to learn and use and does not come at a great expense. This technique is a mix of methods and the client's belief or faith element (centered on a particular manner of expressing God's name or a phrase that has a soothing effect on the client) pronounced in a consistent pattern with acceptance [41].

Researchers must conduct rigorous studies before further generalizing relaxation techniques to replicate their effectiveness and ensure their validity and reliability [42,43]. Researchers have yet to work with RCTs on medicines and various complementary and alternative therapies for PMS. There is an obvious need for more studies on complementary and alternative treatments for PMS. Trials with suitably hefty samples are necessary to reduce type II statistical errors. However, in the search for large samples, standards for ensuring an accurate classification of PMS (mild, moderate, severe) should be considered [38]. Women who meet the PMS criteria depending on retrospective studies should validate symptoms with contemporary everyday evaluations for at least every two cycles before being enrolled [39].

A study conducted by Ferreira and Kulkarni revealed that Mitchell's relaxation techniques were more effective in lowering the severity of PMS, especially fatigue and headaches [23]. In a study by Celik et al., regular practice of progressive relaxation techniques reduced dysmenorrheal discomfort. According to their findings, nurses should advise women suffering from dysmenorrhea to try relaxation techniques and tell them of their advantages [44].

In conclusion, the study conducted by Amirova et al. establishes that Mitchell's method effectively reduces sleep insufficiency, sleep issues, weariness, and discomfort in fibromyalgia patients. This treatment approach only occasionally demands significant time and effort, rendering it a practical and cost-effective method for managing fibromyalgia symptoms in individuals without associated restless leg syndrome (RLS). The research underscores the importance of proper sleep management strategies in fibromyalgia treatment. The absence of side effects, utilization of rigorous evaluation methodologies, and demonstrated efficiency in symptom management distinguish it as a valuable adjunct therapy for individuals with fibromyalgia [45].

A study by Mowla et al. concluded that Benson's relaxation technique helped improve the quality of life of the primary caregivers of children with chronic diseases [37]. According to a study by Poorolajal et al., Benson's treatment is a safe and cost-effective strategy for reducing preoperative anxiety and hemodynamic reactions in general surgery patients [38]. According to Barabady et al.'s findings, Benson's relaxation approach, as a non-pharmacological technique, proves highly effective in reducing preoperative anxiety among cataract surgery patients. Moreover, it significantly diminishes the necessity for propofol, an anesthetic medication, during the surgical procedure. These results underscore the potential of Benson's relaxation approach as a valuable intervention in preoperative care for cataract surgery patients, with the added benefit of potentially reducing the usage of anesthesia medications [46]. An interventional study conducted by Hesmatifar et al. revealed that in hemodialysis patients, Benson's relaxation technique proves beneficial by lowering the levels of depression [39].

The comparative effects of Mitchell's and Benson's relaxation techniques on PMS still need to be explored in the existing literature. Although there is research available on various types of relaxation techniques and their outcomes, a consistent lack of substantial evidence supporting the effects of these techniques on quality of life and pain in women with PMS persists. Therefore, a pressing need exists to examine further literature on the direct comparison between relaxation techniques for managing PMS.

This study underscores the importance of conducting high-quality, evidence-based research to shed light on the potential benefits and efficacy of Mitchell's and Benson's relaxation techniques in alleviating PMS symptoms. Additionally, the study provides valuable evidence of the effects of these relaxation techniques on various diseases and highlights their proven effectiveness in treating anxiety and other psychosocial factors. Nonetheless, the specific impact of these techniques on PMS necessitates further investigation through well-designed and rigorous studies to establish a clear understanding of their potential contributions to PMS management.

Limitations and recommendation

Despite the exhaustive efforts to conduct a comprehensive search for published papers, certain relevant research may have gone unnoticed due to the reliance on specific data sources. Furthermore, the constraints on available resources necessitated limiting the search scope to English-language publications, possibly excluding valuable insights from other languages. The absence of studies focusing specifically on the effects of relaxation techniques in alleviating PMS highlights a distinct and urgent need for further research. Thorough investigations hold immense significance as they are crucial in developing a robust therapeutic intervention protocol to reduce PMS symptoms effectively.

Hence, we recommend undertaking additional research endeavors to comprehensively explore and elucidate the potential benefits of relaxation techniques in managing PMS. By conducting more experimental extensive studies, a profound and holistic understanding of the efficacy of these techniques can be attained, thus paving the way for developing improved and tailored treatment strategies for individuals afflicted with this condition. Such advancements hold promising prospects for significantly enhancing the quality of life and overall well-being of those grappling with PMS.

## Conclusions

In conclusion, the current state of research calls for a pressing need to gather high-quality evidence with reliable outcome measures to support the assertion that relaxation techniques offer optimal alleviation for PMS. Despite the study's findings, which highlight a lack of rigorous investigation into alternative treatments for PMS and the absence of any therapy showing 100% success, it is crucial to subject all putative medicines to well-designed RCTs for a thorough evaluation. Although scientific proof of targeted benefits for PMS is yet to be established for specific complementary/alternative therapies, including relaxation techniques, we can recommend them to promote overall health and consider them reasonably safe. Considering the popularity of complementary/alternative treatments among PMS sufferers, there is an apparent necessity for further research to determine their effectiveness in addressing this condition. As we strive to advance our understanding, rigorous exploration of relaxation techniques holds promise for contributing to enhanced PMS management strategies.
